# Long-term outcomes of adult patients admitted with sepsis to brazilian public hospitals: a national retrospective matched cohort study

**DOI:** 10.1186/2197-425X-3-S1-A86

**Published:** 2015-10-01

**Authors:** LCPd Azevedo, C Toscano, AL Andrade, AL Bierrenbach

**Affiliations:** Hospital Sirio-Libanes, Sao Paulo, Brazil; Federal University of Goias, Goiania, Brazil

## Introduction

Although sepsis outcomes are relatively well known for developed countries, very few studies evaluated the long-term prognosis of septic patients at a national level in developing countries.

## Objective

To determine the magnitude and duration of the effects of sepsis on survival of adult patients hospitalized in public hospitals in Brazil.

## Methods

Retrospective cohort study using the national hospitalization database of patients older than 15 years admitted to public hospitals from 2005 to 2010. These patients were residents in 10 state capital cities comprising all geographic regions of the country. Sepsis episodes were identified according to sepsis-associated International Classification of Diseases codes (ICD-10) [1]. Patients with at least one episode of sepsis during their hospitalizations were matched by year of hospital admission, age group, gender and postal code to patients hospitalized due to other causes. Hospitalization records of sepsis cases and controls were linked through a deterministic linkage to records of the national mortality information system from 2005 to 2011. Kaplan-Meier and Cox regression models were used to evaluate overall and long-term mortality. Long-term mortality was defined as occurring after discharge of the first hospitalization due to sepsis for cases or to other causes for controls.

## Results

**Figure 1:**
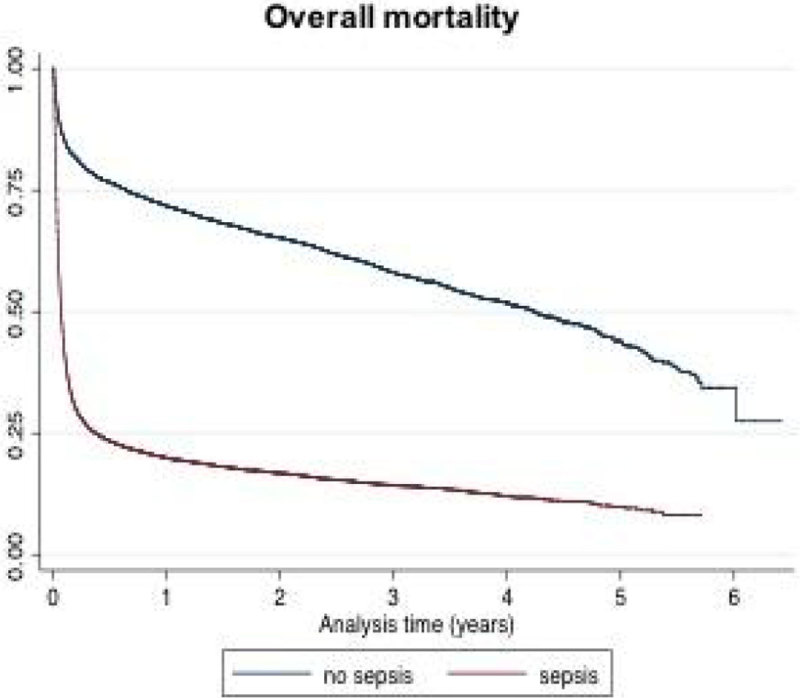
**Overall mortality. Long-term mortality rate was 17.4% for septic patients and 7.9% for controls (p < 0.001), with a hazard ratio of 1.4 (CI95% 1.3-1.5), after adjustment for ICU stay (fig. 2).**

We identified 33,552 patients with at least one episode of sepsis during hospitalizations, which were compared to 33,341 hospitalized controls. Patients were followed for a minimum of one and a maximum of 6 years. ICU admissions at least once during hospitalizations were 43.5% for sepsis cases and 4.6% for controls. Overall mortality rate was 62.1% for septic patients and 13% for controls (p < 0.001), with a hazard ratio of 4.2 (CI95% 4.0-4.3), after adjustment for ICU stay (figure 1).

**Figure 2:**
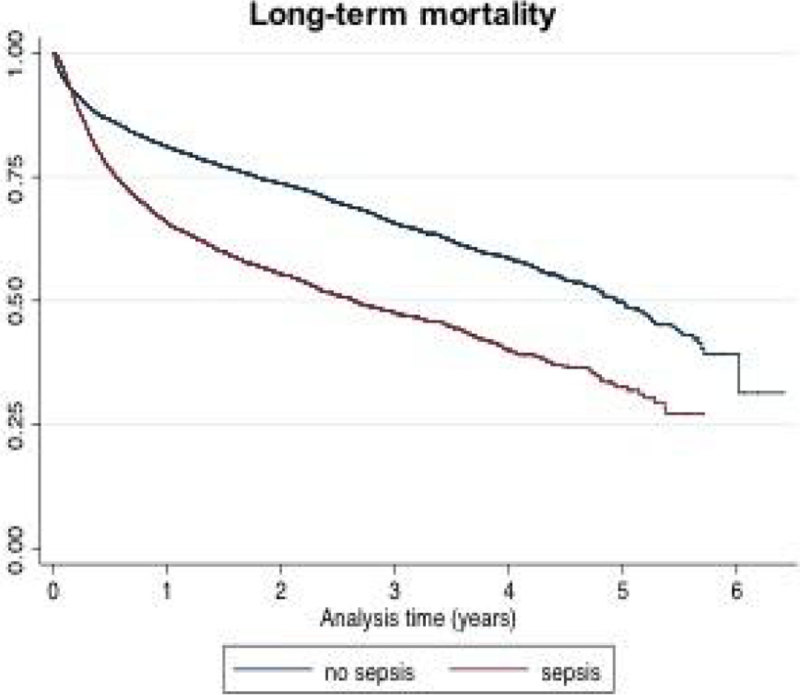
**Long-term mortality ICU stay was a positive confounder, particularly for the overall mortality hazard ratio.**

## Conclusions

This study confirms previous findings of excessive mortality rates for sepsis in Brazil [2]. Moreover, septic patients who survive hospitalization in public hospitals have a higher mortality for subsequent years as compared to matched hospitalized controls.

## References

[1] Taniguchi LU (2014). Crit Care.

[2] Conde KA (2013). PLoS One.

